# Women’s perspectives on caesarean section recovery, infection and the PREPS trial: a qualitative pilot study

**DOI:** 10.1186/s12884-019-2402-8

**Published:** 2019-07-15

**Authors:** Annalise Weckesser, Nicola Farmer, Rinita Dam, Amie Wilson, Victoria Hodgetts Morton, R. Katie Morris

**Affiliations:** 1 Centre for Social Care, Health and Related Research, Birmingham City University, Birmingham, B15 3TN UK; 2 Birmingham Women’s Hospital, Birmingham, B152TG UK; 3 Institute of Metabolism and Systems Research, University of Birmingham, Birmingham, B152TT UK; 4 Institute of Applied Health Research, University of Birmingham, Birmingham, B152TT UK

**Keywords:** Caesarean section, Postnatal infection, Caesarean section recovery, Patient experience, Qualitative research

## Abstract

**Background:**

In England, 27.8% of all pregnant women undergo caesarean sections (CS) to deliver their babies. Women undergoing CS are at risk of developing sepsis and post-natal infections, which not only contribute significantly to maternal mortality and morbidity, but also negatively impact upon post-natal recovery and wellbeing. This study explores patients’ priorities in relation to CS recovery, focusing on their knowledge and experiences of infection prevention. The study formed part of the PREPS (Vaginal Preparation at caesarean section to Reduce Endometritis and Prevent Sepsis – a feasibility study of chlorhexidine) Trial; patients’ views on the PREPS Trial were also sought.

**Methods:**

Using qualitative methodology, two focus groups and six telephone interviews were carried out between September and October 2017 with a total of 21 women who had undergone a CS within the preceding six months. Focus groups and individual telephone interviews were audio-recorded and transcribed verbatim; a thematic analysis was conducted using NVivo 11.

**Results:**

Women’s priorities around CS recovery centred on pain (or the lack thereof), mobility and the ability to resume everyday activities, including caregiving. Those undergoing a CS for the first time reported not feeling confident in their ability to identify signs of infection and sought visiting health professionals’ expertise and reassurance. Women were unable to recall whether they had received information regarding infection prevention and felt that they had not received sufficient advice. Some reported receiving general information regarding CS recovery, which ranged in quality. Prevention of womb infection is a major goal of the PREPS trial, however, the majority of women were not aware that womb (as opposed to wound) infection was a post CS risk.

**Conclusions:**

Women undergoing a CS want more information on what constitutes a ‘normal’ post-operative recovery and specifically would welcome written information and infection prevention advice. This should be a key element of improving post-CS maternal experiences and potentially reducing sepsis and infection rates. CS stigma negatively impacts women’s recovery experiences and possibly information provision.

The PREPS team incorporated findings regarding consent pathways for recruiting women into intrapartum research and developed two patient reported outcomes to collect in the main trial.

**Trial registration:**

The PREPS trial has been registered with ISRCTN on the 10th July 2017 (ISRCTN33435996).

## Background

Caesarean section (CS) is a life-saving surgical intervention performed when complications occur during pregnancy and/or labour and is the most common major operation worldwide [1]. Twenty to 35% of all babies are delivered by CS in high income countries [2] and over a quarter (27.8%) of all babies in England were delivered by CS between 2016 and 2017 [3]. Women undergoing CS are at risk of developing sepsis and postnatal infection [4], which are not only significant contributors to maternal morbidity and mortality but also barriers to postnatal recovery and maternal wellbeing.

The most common types of infections following CS delivery include, postpartum endometritis (womb infection) [5, 6] and skin incision (wound) infections, which are both components of a surgical site infections (SSIs) according to US Centres for Disease Control and Prevention definitions [7–10]. One England-based cohort study found that nearly 10% of all women undergoing a CS developed postsurgical infections [8], rates higher than those of other comparable operations such as hysterectomies. Sepsis reduction, SSIs reduction and reducing antibiotic use have been identified as national [11–13] and international [14–16] priorities. Reduction in these complications will improve maternal health and potentially neonatal wellbeing through the facilitation of ongoing breastfeeding and the reduction of interference with the attachment processes.

The PREPS trial (Vaginal Preparation at caesarean section to Reduce Endometritis and Prevent Sepsis – a feasibility study of chlorhexidine) is a feasibility study investigating whether cleansing the vagina immediately before CS, with the antiseptic solution Chlorhexidine, will reduce the risk of maternal postnatal endometritis and sepsis [17]. The aims of the qualitative component of the PREPS trial are two-fold. First, the study seeks to understand women’s priorities in relation to CS recovery and infection prevention. Additionally, it aims to explore women’s views on vaginal cleansing and randomisation into such a trial, specifically focussing on consenting to the trial in labour and potentially in an emergency scenario.

There is a paucity of qualitative research on women’s experiences of undergoing a CS [18–22]. Even fewer such studies focus on CS recovery [23, 24] and none focus on CS postoperative infection and infection prevention. Women report that their physical recovery after CS was hindered by a range of health issues, including pain and reduced mobility, abdominal wound problems, infection, vaginal bleeding and urinary incontinence [23]. They also report experiencing extreme pain in the postpartum period, hindering their mobility and ability to carry out normal day-to-day activities [19, 21, 22, 24] including difficulties in breastfeeding [25]. Many were “not ready for the intensity and duration of the postoperative pain” of physical recovery ( [19] – p.45). In relation to infection, one study found women had a heightened awareness of postoperative infection [24] and women found scarring after wound infection to be unsightly and disfiguring [23].

Women report negative experiences regarding the quality of care that they received before and after caesarean birth [24] and feel judged by health care practitioners when having elective CS [18, 19]. Feelings of failure as a mother were experienced and explained in relation to practical difficulties in caring for new-born babies as a result of women’s physical recovery from CS [19]. Similarly, feelings of guilt were experienced when women felt their ability to take care of their newborn baby was limited by postoperative complications and their need for medical attention [23]. New mothers have been found to perform the majority of caring work, including care of the home and of older children and this reality often conflicts with postoperative advice on the need to rest [23].

Research suggests the need for a different approach to the provision of information about recovery. Postoperative advice was found to conflict with what was important to women, with women themselves denying the extent of their physical activity [23]. In addition, women need more detailed information, more of their questions answered, their level of knowledge and understanding to be assessed, and space to be made for a discussion of events after birth [18, 19]. This study begins to help address the paucity of qualitative evidence on women’s own priorities and information needs in relation to CS recovery and infection prevention.

## Methods

As this was an exploration of CS recovery and infection (prevention) as experienced by women themselves, a qualitative research methodology was used. Telephone interviews and focus group discussions were employed. This study was carried out in a maternity hospital in the West Midlands. Data was collected between September and October of 2017.

A preliminary literature review of qualitative studies on women’s experiences of CS recovery was conducted. The focus group and telephone interview topic guides were informed by themes identified in this review. The topic guides were also reviewed and informed by the expertise of the PREPS Trial Management Group.

Two focus groups and six telephone interviews were carried out with women who had undergone caesarean sections in the preceding six months. Focus groups were conducted to generate data that capitalises on the communication between research participants [26]. Telephone interviews offered a flexible means to include women with new-born babies and thus for whom attending a focus group at the hospital was a challenge [27].

Ethical approval and permission for this study was granted from the London City & East Research Ethics Committee (17/LO/0874) and local approval was granted from Birmingham Women’s Hospital. NF, a research midwife, recruited and obtained written informed consent from all participants, by approaching women on the postnatal wards at the recruiting hospital and through social media adverts. The study employed purposive sampling, recruiting participants with characteristics and experiences relevant to the research question. At the beginning of each focus group and telephone interview, consent to record interviews was verbally reconfirmed. The qualitative component of PREPS was conducted prior to the randomised controlled trial to allow information obtained to feed into trial design, processes and outcomes. Therefore, women in the qualitative study were not enrolled in, nor recruited to the randomisation feasibility trial.

AWi, a senior qualitative health researcher, facilitated the focus groups with NF observing, taking field notes, and providing further information and supports for participants if required. AWi and RD, a research assistant, carried out telephone interviews. AWi and RD are trained, experienced qualitative researchers with expertise in gendered experiences of reproductive health. AWi, RD and NF are female, matching the gender of the research participants. There was no established relationship between the researchers and the participants prior to the study. AWi and RD introduced themselves as researchers from the Birmingham City University and NF introduced herself as a research midwife from Birmingham Women’s Hospital to participants prior to focus groups and/or telephone interviews. Each focus group discussion lasted approximately one hour, and telephone interviews took between 20 to 30 min each. Focus groups were held in meeting rooms within the participating hospital. As an expression of appreciation for their time, all participants received £15 vouchers after focus groups/telephone interviews. Focus group participants were additionally reimbursed for parking. Focus groups and interviews were audio digitally recorded and transcribed verbatim.

Thematic analysis [28] was carried out by the qualitative research team (AWi and RD). To establish trustworthiness [29], AWi and RD independently read the transcripts line by line, identifying emergent themes and created initial codes. VHM also read transcriptions and provided clinical insights to the analysis. AWi and RD brought codes together to create a coding framework, and RD coded the transcripts with NVIVO 11. RD employed constant comparison, an iterative method of analysis, searching for each themed code throughout the entire data set (focus group and telephone interview data), comparing all instances until no new themes were identified. Saturation was deemed to have been reached when no new themes were emerging from additional interview data. AWi and RD discussed and agreed upon common patterns and broader themes from women’s perspectives on CS recovery and infection (prevention). Dissident views and areas of diversity were considered. Generalisation is not an aim of qualitative research; however, analysis findings are consistent with the existing literature on women’s experience of CSs.

## Results

A total of 21 participants took part in the study; six took part in telephone interviews and 15 in focus groups (eight in Group A and seven in Group B). Participants ranged in age (from 26 to 45 years); for over half (*n* = 12), this was their first CS, with the other 9 participants having 1 to 3 CSs previously. For over half (*n* = 12), their previous CS was elective, whilst 9 participants had had emergency CSs. Fosur participants reported experiences of infection; two related to wound haematomas, one had endometritis and one superficial wound infection. Of those experiencing infection, three were readmitted to hospital and one went to their GP.

A summary of sample characteristics is provided in Table 1*.*

**Table 1:** Characteristics of sample

Sample (*N* = 21)				
Age (years)	Range = 26–45	Mean = 34.4		
Marital Status	Married = 15	Partner = 5	No response = 1	
Ethnicity	White British = 16	British Asian = 1	Mixed race British = 2	White American = 1 African =1, Asian =1
Employment	Yes = 19	No = 2		
Contracted employment hours	Full time = 11	Part-time = 8	Unemployed = 2	
No. of children	Range = 1–4	Mean = 1.9		
First CS	Yes = 12	No = 9		
No. of CSs in the past	None = 12	1–3 = 9		
Type of CS	Elective = 12	Emergency = 9		
Experience of infection after last CS	Yes = 4	No = 17		

The thematic findings presented here derive from the combined analysis of focus groups and telephone interviews.

### Experiences of pain

Women’s descriptions of CS recovery centred on experiences of pain or lack thereof. Women who had underwent their first CS reported varied experiences with pain. For some, the severity of pain was unexpected and the prescribed pain medication insufficient:

*[T]he first three weeks…three or four weeks, it was like really painful and sore. I couldn’t even bend* [Interview #3]

*[My CS] was an emergency, so I wasn’t expecting it. They gave me painkillers to go home that didn’t cut it really… I thought it was going to be painful, but I did get a bit of a shock.* [Focus Group A]

*The painkillers are wildly inadequate. I went to my GP the same day I came out [of hospital], just [for them] to say, you could still have a double dose of Ibuprofen.* [Focus Group A]

One woman normalised the extreme pain she had experienced (pain caused by an undiagnosed haematoma), stating that she ‘just thought everybody’ who underwent CSs had similarly ‘excruciating pain.’ In contrast, others reported less experiences of physical pain after their first CS:

*Mine has actually been excellent, I was absolutely fine* [in relation to pain], *but I had a really good wound. I hardly bled.* [Interviewee #4]

I didn’t have much pain, so mine’s been okay. The first 24 hours I found it really uncomfortable. [Then] I got up, started moving around and it wasn’t so bad after that*.* [Focus Group A]

Those who had had more than one CS previously, compared their different CS recovery experiences in relation to pain or lack thereof:

*This time it’s gone really quick, I found it a lot easier knowing what I should be doing. Last time it was just very painful and took me a lot longer to recover. [Focus Group A]*

*The first time round was probably more painful than the second time round, but the second time round after a week it was fine…I was more mobile. I think it was a good experience. [Focus Group B]*

Discussions of experiences of pain centred on its severity, duration and the ways in which it impacted upon women’s ability to ‘get up and about.’ A ‘good’ recovery was felt to be one where a woman was ‘out of pain’ within a few weeks and able to resume ‘normal life’ not too long after.

### Mobility, caregiving and everyday activities

Women measured their recovery progress not only on pain cessation, but also in terms of their ability to carry out caregiving activities (lifting babies, breastfeeding and doing night feeds) and resume everyday activities, such as going for walks and driving.

*I couldn't lie down at night because there was like a dragging and pulling feeling, which was very, very painful. I couldn't move at one point, I had to get my husband to help, which isn't great if you're feeding in the night, et cetera.* [Interview #5]

*I was pretty mobile straight away; I went up and down the stairs straight away*. [Interview #4]

*By two weeks I felt, kind of, back to myself. But I have, like, little twinges, obviously. I’d still have to be cautious, but, I was back driving and was okay then after that*. [Focus Group B]

Some women felt that there is ‘almost a competition’ between new mothers who have had CS deliveries vs vaginal deliveries, who compare not only who had ‘the most horrendous’ labour experiences but also who regains mobility most quickly. One woman stated:

I do feel like you have to justify the reasons why you’ve had [a CS]. [Interview #1]

In addition to feeling she must ‘justify’ her need for operation an operation, she felt pressure to quickly begin (re)engaging in activities (attending baby play groups, social coffees and park walks) with other ‘mum friends’ who had had ‘natural births’ so that ‘they could see I was up and about and I’d got it all under control.’

### Infection: experiences, knowledge and health seeking

Four participants had experienced a CS related infection. For them, the ways in which infection prolonged their overall recovery time was a key concern. Women sought care if their wound changed colour, their wound did not close and the consistency of blood changed, and/or if they had ‘the shakes’ and an elevated temperature. A woman who required treatment for a haematoma related wound infection found returning to hospital highly undesirable, she states:

*I didn’t want to go back, but I thought maybe I should because I suddenly felt quite ill… [W]hen [hospital staff] said about going back to theatre, that was that, I’m not coming back to hospital, not when I made it home, no way.* [Focus Group A]

CS related infection was a concern during recovery; a concern that some women reported feeling anxious about. Those who had never had CSs before reported uncertainty in their ability to identify the signs of infection:

*I got really paranoid that I’d caused an infection for some reason. So I had this little mirror that I would look at the wound with all the time and go “What’s it doing? Is it getting dryer?” I’m not normally like that, not over-anxious.* [Focus Group B]

*My concern was, would I know if I had an infection.* [Interview #6]

*[T]here’s probably some people that wouldn’t recognise the signs of an infection. [It is a problem] if you’ve got no medical professional wanting to follow-up and have a look.* [Interview #1]

When women suspected that their wound was infected and/or not healing, they sought reassurance from home-visiting midwives or nurses. They report mixed experiences when asking visiting health professionals to check their CS wound:

*I said to the midwife I had some concerns about the wound… [I]t was weeping quite a lot, so I said I was a bit worried about it and she said talk to the practice nurse, because they’re usually the experts in wound care.* [Focus Group A]

*They wouldn’t check your scar unless you said “Oh, I’m a bit concerned” or “Can you just have a look at it?” I’ve still got it now, there’s like a really delicate piece of skin which is really pink…but it just looks and feels delicate, but really thin. And it worries me… Everytime she’d come I’d say can you just look at it and make sure it’s okay, but if you didn’task she wouldn't check my scar.* [Focus Group B]

*I think the midwives are really good at checking [wounds], and [my wound] was normalwhen my midwife looked at it. It just then got infected afterwards. They have to give the responsibility to you to look at it every day. But you google pictures of normal c-section scars and I was like [my wound] doesn’t look like that.* [Focus Group B]

Women recognised their role in identifying signs of infection, as well as in general wound care. The internet was a source of information for some, however, most sought the expertise of a midwife or nurse when uncertain about signs of infection or how their wound was healing. While some were satisfied with the care they received, those who were not felt that health professionals’ focus on care for new-born babies caused them to neglect care of new mothers. During home visits, enquiries about a women’s’ recovery were seen, by some, to come only at the end, ‘*It’s like in the last 30 seconds, “How are you? How’s the scar? See you next week.”’*

Discussions of infection overwhelmingly focused on wound healing and care. The majority of participants were not aware of the risk and signs of endometritis (excessive bleeding, prolonged pain, which left untreated can lead to sepsis). One participant experienced endometritis, but others who reported experiences of excessive bleeding did not identify this as a possible sign of infection.

### Infection: information provision and needs

Some women reported being unable to recall whether they had received information about preventing infection:

*I don’t remember specifically talking about infections or signs of infection. I’m sure they did probably go through it, you know, as one of those things that they sort of talk about as a risk.* [Interview #2]

*Maybe I was given the information, but I was too tired at the time to listen, because I had the new baby and I had been in labour and awake for four days, then I had the [emergency] C-section… I think you’re that tired that you don’t necessarily process everything until afterwards.* [Focus Group A]

Many women did not recall being given information regarding CS recovery and infection prevention, especially if such information was given shortly after operations. Those undergoing elective CSs recall being given paperwork regarding associated risks to them and their babies, but did not recall receiving medical advice about how to prevent infection post CS. Others, however, recalled being given some CS recovery advice, focused on wound care:

*The midwife would come round and they obviously told me how to keep it clean and how long it would take to heal and they told me what I should and shouldn’t do… [*Interview #6]

*The midwife gives you that form at the end when you leave the hospital and it says if this happens to your scar, go here, phone this person.* [Focus Group B]

*[Midwives talked] about bathing [the wound], not rubbing it, just patting it dry and keepingit clean, airing it. I think those were the general things and to look out for any signs of, likeany pus or anything like that.* [Interview #1]

One woman felt that she was not given adequate information about CS recovery and infection because her health team ‘judged’ her for having an elective CS delivery:

*When [my partner and I] elected to have the caesarean you could just tell by [the midwife’s] demeanour, she did not agree with it… We wanted someone to give us the facts both sides, not really to air their opinion.* [Focus Group B]

Other participants reported receiving an inadequate amount of information (compared to other comparable forms of surgery) or only receiving information after contracting an infection or after engaging in an activity that hampered recovery (e.g. lifting baby from cot):

*My mum had a hysterectomy and the level of information she got for a fairly similar surgery was mountains and mountains. And we just like, don’t have anything…* [Focus Group A]

*I’ve had major surgery in the past and there’s a follow-up after, but with sections, I don’t find that.* [Interview 1]

*The [community midwives] showed me how to feed safely and gave me some tips, because I wasn’t told [prior]. I wasn’t told about showering or having a bath and I don’t know whether having a bath straight away helped [contribute to] the infection or whether it was because [the baby] was kicking me.* [Focus Group A]

*[On the postnatal ward] they said to me on about day three to buzzer if I wanted to get the baby out of the cot, and I was like oh, nobody had ever said to me don’t get the baby out yourself.* [Focus Group B]

In addition to more information regarding infection prevention, women also wanted information regarding how to position breastfeeding babies to protect CSs wounds, and advice warning that one may feel ‘okay’ and ‘healed,’ but not to prematurely resume activities such as hoovering or driving as this could cause the wound to reopen. Women identified ways in which information provision could be improved. Information given post-surgery was difficult to recall and thus advised that this was better to receive in the form of a pamphlet. One woman, felt too much paperwork was already given and worried this information would get lost. Pamphlets should not only include information about wound care and identifying signs of infection, but specific information on who to contact should one suspect infection. While some participants report receiving such a pamphlet, not all did. One participant felt that standard antenatal classes do not provide enough information about CS and CS recovery as these courses primarily focus on ‘natural birth.’

### Views on PREPS trial

*Women understood the purposes of carrying out a randomised controlled trial (RCT),but expressed confusion about the purpose of the PREPS trial –* specifically the pathogenic mechanism of cleansing the vagina to reduce endometritis*. However, once the rationale was understood the participants thought the PREPS Trial was a worthwhile one:*

*I don’t see how cleansing someone’s vagina when they cut through layers of your womb – how that is going to help reduce an infection, it just seems, too far away.* [Focus Group A]

*I thought it was a very valid trial. If I’m honest, I didn’t realise that there would be an internal infection. I was just thinking about wound incision infection.* [Interview #5]

*The gel [for vaginal cleansing] is a good idea, yeah, ‘cause infections are not very nice…You don’t think about it like that though. You just think of your belly at the time.* [Interview #3]

A majority reported that they would participate in the PREPS Trial if asked for reasons of altruism (i.e. to assist other women undergoing CS in the future and research that could lead to reduction in infection rates) and because the trial was not seen to pose any risks to them or their babies. Women found randomisation into one of the two trial wings (stomach cleanse, or stomach and vaginal cleanse) acceptable, as one would receive either the standard practice of care (stomach cleanse) or the standard practice plus the additional safeguard of a vaginal cleanse. Women believed the success of the RCT would depend on careful explanation of the trial to potential recruits:

*You either get the standard care [stomach cleanse] or the upgrade [stomach and vaginal cleanse]… Some people might think, “Well why aren’t we all getting the better thing anyway?” I guess it would need to be explained quite carefully, wouldn’t it?* [Interview #2]

*I’d probably want to know exactly what the procedure would involve, because I find it fairly mortifying having the whole drape and catheter in and just being aware I wasnaked in a room with five people looking at me.* [Focus Group A]

While feeling the PREPS trial is a worthwhile one, and that randomisation is acceptable as vaginal cleansing was perceived as an ‘upgrade’ to standard practice, some believed potential recruits to the RCT would require detailed information about the reason for randomisation and preoperative cleansing procedures.

Women were asked about the pathway of consenting participants undergoing emergency CS to the RCT. The PREPS trial team had developed a proposed pathway of consent for an emergency where women would be provided with written information about the trial in the antenatal period and the during the intrapartum period if appropriate. In an emergency women would then be asked to give verbal consent for the randomisation and intervention with written consent and further information following the emergency CS. They reported that recruitment was acceptable as long as verbal pre-operative information provided was brief, with full information given post-operation and written consent obtained at this point:

*[P]patients can just say “Okay, in principle I agree, but I will discuss it in more detail later.” And then obviously after they’ll sign a full consent form having read the full information to say that they’re happy to continue with information collection.* [Focus Group B]

*If like the bare basics of [the trial are explained] just briefly – because you won’t be thinking straight, you, like, you won’t care – and personally I would [participate*]. [Focus Group A]

## Discussion

The aim of this study was to explore women’s priorities in relation to CS recovery and infection, as well as their views on the PREPS Trial. Findings from the study provide important new insights into women’s experiences and knowledge of CS infection prevention and begin to help address the dearth of qualitative data on CS recovery. Infection and sepsis are common and are directly linked to maternal morbidity and mortality, further research and improvement is needed on how information and which information, is provided to women to allow timely and appropriate identification of infection.

Findings centred around women’s experiences of pain, (im)mobility, the ability to carry out every day/caregiving activities are consistent with previous studies on CS recovery [23, 24] and general experiences [19–22]. A 2013 meta-synthesis of research on CS experiences [19] found that women felt unprepared for the intensity and duration of post-operative pain they experienced. Similarly, this study found women who had undergone a CS for the first time were surprised and ‘shocked’ by the level of pain experienced and felt the initial pain medication received was inadequate. Additionally, this study shows that those who have had multiple CSs, understand and measure the quality of their recoveries in terms of relative, comparative pain (or lack thereof) after each CS operation. Thus, pain, its severity and duration, is a key priority in women’s own understandings of what constitutes a positive or negative CS recovery experience.

Previous research demonstrates that pain in the postpartum period hinders women’s mobility and ability to carry out their normal day-to-day activities [19, 21, 22, 24]. This study also found that the ability to resume ‘normal life’ and every day activities (including driving and going for walks) to be a priority of women in CS recovery. The ability to lift infants, feed babies independently at night and position a breastfeeding baby whilst protecting one’s wound were also concerns. Qualitative [25] and quantitative [30] studies on breastfeeding challenges post CS delivery also found maternal mobility limitations and positioning difficulties to be breastfeeding obstacles.

A 2010 qualitative study on CS care [24] showed that women have a heightened awareness of CS related infection. This study found that not only do some women feel anxious about contracting an infection, but they also feel uncertain about their ability to identify signs of infection and seek the expertise of visiting health professionals for reassurance when they suspect infection and/or their wound is not healing. There is confusion about which visiting health professionals (midwives or nurses) should check women’s wounds and whether it is standard practice to check wounds or to only check upon women’s request. Additionally, there is evidence that some women may normalise post CS pain experiences, which could delay care seeking.

Women’s knowledge of and concerns about CS infection centre primarily on wound infection and scarring; similar findings arose in Kealy, Small and Liamputtong’s 2010 study [23]. This study additionally demonstrates that the occurrence of endometritis and its associated signs are not widely known. Lack of awareness about the occurrence of endometritis was also evident in women’s views of the PREPS Trial; with women seeing the RCT as a worthwhile and valuable one once understanding how vaginal cleansing may prevent endometritis.

Previous research suggests the need for a different approach to the provision of information about CS recovery [19, 23], with women needing their level of knowledge assessed, more detailed information, more of their questions answered, and more time with visiting health professionals for discussions regarding recovery [19]. This study confirms such needs, with women: having varied information needs depending on the number of CS operations they have had previously; wanting more information regarding CS recovery and infection prevention – information comparable to that provided with other similar operations; wanting improved communication with visiting health professionals regarding their recovery and wound healing. Additionally, this study provides valuable insights into women’s information provision needs in relation to infection prevention. Women need more detailed (and written) information about wound and womb infection (prevention), signs of infection, and clearer guidance on who to contact should they suspect infection.

Tully and Ball, based on their 2013 large-scale qualitative study on women’s explanations for and experiences of CS deliveries [22], report that women spontaneously defended themselves against the stigma of the ‘too posh to push’ label, with women discussing the appropriateness of CS as a social critique rather than a health issue. This study found that such stigma and judgement also shapes women’s recovery experiences and possibly information provision. Women reported feeling in an unspoken ‘competition’ with women who had had vaginal deliveries and that they must ‘justify’ the need for their CS delivery and must ‘keep up’ with the recovery pace of peers who have had vaginal deliveries. A participant believed that health practitioners’ judgement influenced why they received little to no information regarding CS recovery and infection prevention during antenatal classes, or pre- or post-operation. While only one participant reported such feelings, two previous studies have similarly found women experience judgement from health practitioners for undergoing a CS [18, 19]. Providing further evidence for the recent call from the Royal College of Midwives (RCM) to stop the campaign for and language of ‘normal births’ as it unintentionally reinforces women’s feelings of failure and judgement when needing medical intervention.

### Implications for PREPS trial

To facilitate recruitment of women undergoing emergency CS a verbal consent process was developed by the PREPS trial management group, informed by the discussions within the focus groups. In brief the pathway comprised of all women receiving information prior to labour in the antenatal period. Information was further provided on the labour ward and if necessary and verbal consent was obtained with written consent after the CS.

Women in the PREPS trial were followed up at 14/30 days via telephone interviews the preferred question response by the women was a descriptive option such as ‘a little’ rather than a 0–10 scale. For this interview process patient reported outcomes were also developed and collected.

### Strengths and limitations

To our knowledge, this is the first qualitative study to focus on women’s experiences of CS recovery in relation to infection and infection prevention. While the study sample size is relatively small, the use of two research methods has increased methodological rigour. It is often challenging to engage women who have just given birth and are caring for new-borns in research. Thus, while findings are limited by the small sample size, they are valuable as the views of such women are often difficult to obtain.

### Implications for future research and policy

#### Promising recommendations

We have been able to gain important information on the acceptability of consent for research in labour. This has been translated to a verbal consent pathway that has been tested within the PREPS main trial and has been extremely successful.

#### Additional research

Women reported uncertainty in their knowledge about constituted a ‘typical’ and ‘normal’ caesarean recovery experience, and some did not feel well equipped to identify signs of infection. While some reported receiving information regarding post-surgery care and infection prevention, others stated that they did not receive such information or received it at a time (post-surgery) when they were not able to absorb and remember it. Additional larger-scale qualitative research on how to best to meet a diversity of women’s care, support and information needs in relation to CS recovery and infection prevention is needed. Women’s experiences of stigma and judgement for undergoing a CS, and its impact on care and recovery trajectories, also warrants further investigation.

## Conclusions

We have identified the lack of knowledge about the recovery process following a CS and the need for increased awareness of signs and symptoms of infections. This is important so that women can seek help appropriately should they develop complications. This should be incorporated into routine post-natal education and information sharing.

Women want to take part in research and feel that intrapartum consent is acceptable, verbal consent with written consent after the procedure is appropriate and acceptable.

## Data Availability

To protect participant anonymity, datasets are not publically available. Anonymised datasets analysed in this study, however, are available from Dr. Annalise Weckesser upon reasonable request.

## Abbreviations

CDC: US Centres for Disease Control and Prevention
CS: Caesarean Section
PREPS Trial: Vaginal Preparation at caesarean section to Reduce Endometritis and Prevent Sepsis – a feasibility study of chlorhexidine gluconate
RCT: Randomised Control Trial
